# High frequencies of Non Allelic Homologous Recombination (NAHR) events at the AZF loci and male infertility risk in Indian men

**DOI:** 10.1038/s41598-019-42690-0

**Published:** 2019-04-18

**Authors:** Deepa Selvi Rani, Singh Rajender, Kadupu Pavani, Gyaneshwer Chaubey, Avinash A. Rasalkar, Nalini J. Gupta, Mamta Deendayal, Baidyanath Chakravarty, Kumarasamy Thangaraj

**Affiliations:** 1 CSIR-Centre for Cellular and Molecular Biology, Hyderabad, India; 2 CSIR-Central Drug Research Institute, Lucknow, India; 3 Department of Zoology, Banaras Hindu University, Varanasi, India; 4 Institute of Reproductive Medicine, Salt Lake, Kolkata, India; 5 Infertility Institute and Research Centre, Hyderabad, India

**Keywords:** Genetic testing, Infertility

## Abstract

Deletions in the AZoospermia Factor (AZF) regions (spermatogenesis loci) on the human Y chromosome are reported as one of the most common causes of severe testiculopathy and spermatogenic defects leading to male infertility, yet not much data is available for Indian infertile men. Therefore, we screened for AZF region deletions in 973 infertile men consisting of 771 azoospermia, 105 oligozoospermia and 97 oligoteratozoospermia cases, along with 587 fertile normozoospermic men. The deletion screening was carried out using AZF-specific markers: STSs (Sequence Tagged Sites), SNVs (Single Nucleotide Variations), PCR-RFLP (Polymerase Chain Reaction - Restriction Fragment Length Polymorphism) analysis of STS amplicons, DNA sequencing and Southern hybridization techniques. Our study revealed deletion events in a total of 29.4% of infertile Indian men. Of these, non-allelic homologous recombination (NAHR) events accounted for 25.8%, which included 3.5% AZFb deletions, 2.3% AZFbc deletions, 6.9% complete AZFc deletions, and 13.1% partial AZFc deletions. We observed 3.2% AZFa deletions and a rare long AZFabc region deletion in 0.5% azoospermic men. This study illustrates how the ethnicity, endogamy and long-time geographical isolation of Indian populations might have played a major role in the high frequencies of deletion events.

## Introduction

Microdeletions of the AZFa, AZFb and AZFc regions on the Y chromosome are frequent genetic causes of severe testiculopathy and spermatogenic defects leading to male infertility^1–6^. The deletion of AZFa removes a 0.792 Mb region, which includes two single-copy candidate genes *USP9Y* (*DFFRY*) and *DDX3Y* (*DBY*), is found to have phenotypical consequences in the male germline^7–12^. The AZFb region contains palindromes P2 to P5, as well as the proximal part of P1. The deletion of AZFb originates from homologous recombination between the palindromes P5/proximal P1, which removes a 6.2 Mb fragment^13^ along with multiple copies of the genes *CDY2, EIF1AY, PRY, RBMY1, SMCY, TTY5, TTY6* and is reported to result in abnormal spermatogenesis^14^. The deletion of the combined AZFbc regions (P4/distal P1 and P5/distal P1) removes 24 genes, most of which are present in multiple copies, and has been associated with male fertility^12^.

Homologous recombination between b2 and b4 deletes the complete AZFc region of 3.5 Mb, including the genes *BPY2, CDY1, CSPG4LY, DAZ, GOLGA2LY, TTY3, TTY4*, in variable numbers of copies, and results in spermatogenic failure and male infertility^15,16^. AZFc deletions (including partial AZFc) are more common, because of non-allelic homologous recombination (NAHR) events occurring between the highly homologous repeated sequences (same orientation) present in the AZFc region^12–16^. As a result, the deletion of AZFc may cause spermatogenic failure leading to azoospermia/severe oligozoospermia/oligoteratozoospermia^3,12,13,15–18^. Two candidate genes, *DAZ* - deleted in azoospermia (*DAZ*; 400003), and *CDY1* – chromodomain Y (*CDY1;* 400016) in the AZFc region are critical and required for spermatogenesis and their associations with male infertility are well-studied^19–22^. The *DAZ* gene family consists of 4 copies in two clusters of doublets; cluster I contains *DAZ*1*/DAZ2* and the cluster II contains *DAZ3/DAZ4*^23^, which all encode putative RNA-binding proteins. The *CDY1* gene family consists of 2 functional copies, one within the *DAZ* cluster (*CDY1a*) and the other at the distal end of the *DAZ* cluster *(CDY1b)*^24^. Four major deletion combinations of these two genes (*DAZ1/DAZ2* + *CDY1a*, *DAZ1/DAZ2* + *CDY1b*, *DAZ3/DAZ4* + *CDY1a* and *DAZ3/DAZ4* + *CDY1b*) were reported^25^.

The two partial deletions of the AZFc region, namely gr/gr and b1/b3, both remove ~1.6 Mb of the AZFc region. The gr/gr deletion is identified by the deletion of the STS marker sY1291^15^, and the b1/b3 deletion is identified by the absence of additional STS markers sY1191, sY1197, sY1161 and sY1291^15,16^. Both the gr/gr and b1/b3 deletions retain two copies of the *DAZ* genes^15,16,26–34^. The gr/gr deletion is the most common deletion type, and is caused by recombination events between the amplicons g1-r1-r2 and g2-r3-r4^15,25,35^. The two other types of partial deletions, which result from inversions followed by gr/gr deletions or vice versa, are b2/b3 and b3/b4^15,25^. The b3/b4 followed by gr/gr deletion also removes a 1.6 Mb segment of AZFc but differs in breakpoints^25^. The b2/b3 deletion removes a 1.8 Mb segment of AZFc, which is identified by the deletion of the STS marker sY1191^15^. Since the b2/b3 deletion is larger than the gr/gr deletion, it may increase the risk of complete AZFc deletion^15,25^.

Some studies have also reported other rare deletion patterns, proving that the AZFc segment is highly polymorphic^36,37^. In addition to deletion events, a few duplication events that generate Y chromosome variants with six or eight *DAZ* copies in the AZFc region have also been reported^15,16,38,39^. Partial deletions of the AZFc region are common and have been extensively studied^15,16,26–33^. However, the impact of all such partial AZFc deletions on male infertility is still a matter of debate. Some studies have shown that gr/gr deletions are fixed on specific haplogroup backgrounds using major bi-allelic markers^40,41^ and these backgrounds may thus play a protective role in spermatogenesis^15,37,42^. Some case/control studies have reported significant biases in distribution of haplogroups indicating that a particular haplogroup was at higher risk for infertility^43,44^. However, these association studies lack homogeneity due to the geographical origin/environmental factors or of small sample size^45^.

Although several studies have reported that the deletion of AZF regions on Y chromosome is associated with male infertility, there is no comprehensive study to correlate the deletion of AZF regions with infertility among Indian men. Genetic isolation and endogamy, which are widespread in Indian populations, can play major roles in introducing novel causal variations. Therefore, we undertook the present study to test the following hypotheses: a) whether deletion events of AZF regions on the Y chromosome in the diverse Indian population are associated with infertility; b) if partial deletions of the AZFc region are risk factors for spermatogenic failure among idiopathic infertile India men; c) whether the AZFc partial deletions associated with spermatogenic defects are due of lack of *DAZ* and *CDY1* copies; and d) if any specific Y chromosome haplogroup is associated with any type of AZF deletion type and infertility.

## Results

### Microdeletions of AZFa, AZFb and AZFc regions on the Y chromosome

**Figure 1:**
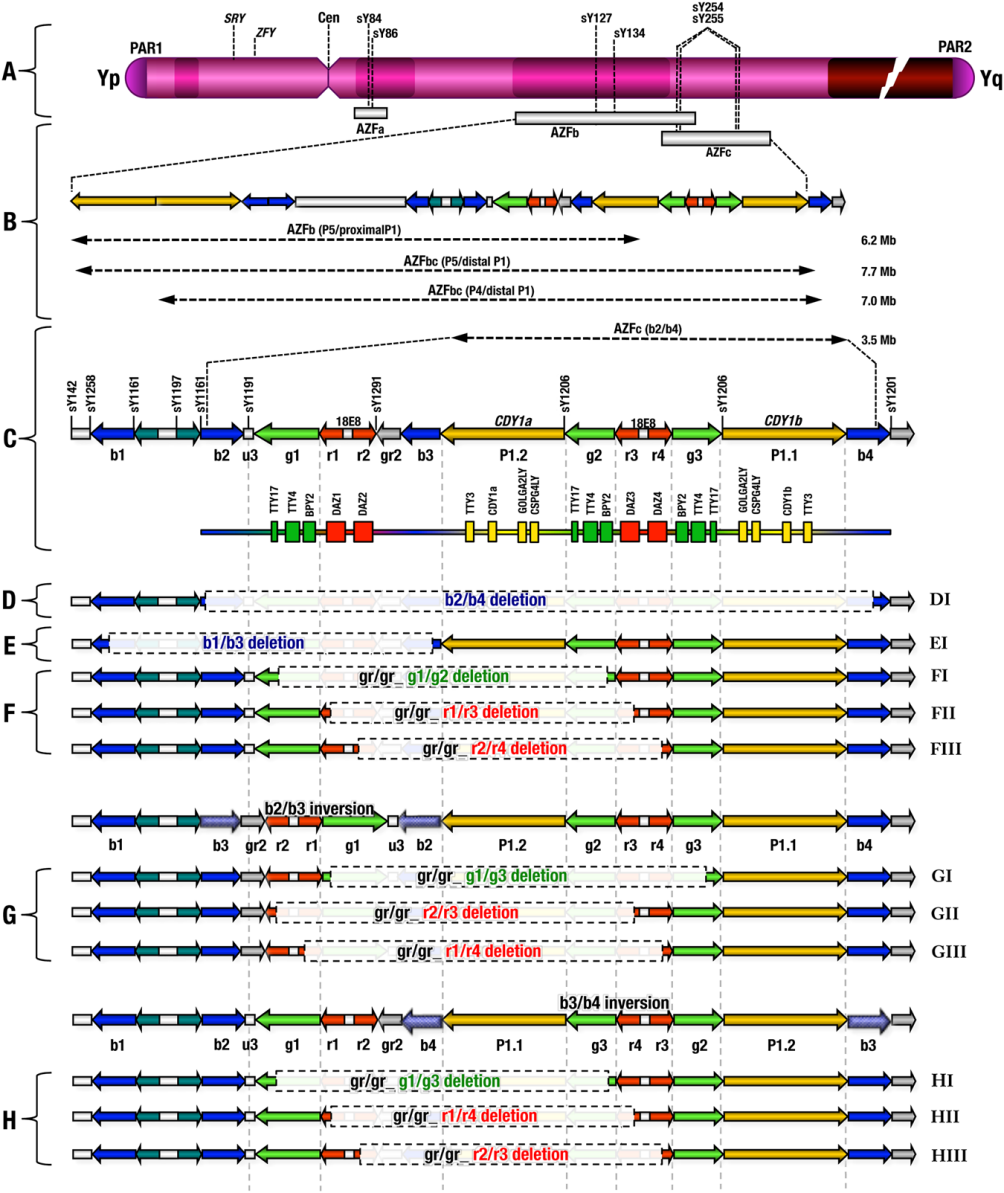
(**A**) Schematic representation of the human Y chromosome structure. (**B**) Schematic diagram showing NAHR events between the P5/proximal P1, P4/distal P1, P5/distal P1 and b2/b4 amplicons. (**C**) Schematic diagram showing the location of direct and inverted repeat sequences, transcription units and STSs markers of the AZFc region, containing the genes and transcription units *BPY2, CDY1, CSPG4LY, DAZ, GOLGA2LY, TTY3* and *TTY4* in variable numbers of copies. (**D**) Schematic picture showing the complete deletion of AZFc results from recombination between the b2 and b4 amplicons, which removes 3.5 Mb of DNA. (**E**) Schematic diagram showing the b1/b3 deletion removes a ~1.6 Mb segment of the AZFc region. (**F**) Schematic diagram showing the gr/gr deletion, it is the most common deletion type caused by the recombination events between the amplicons g1-r1-r2 and g2-r3-r4. (**G**) Schematic diagram showing the b2/b3 inversion followed by gr/gr deletion or vice versa remove 1.8 Mb segment of AZFc region. (**H**) Schematic diagram showing the b3/b4 inversion followed by gr/gr deletion, or vice versa, removes a ~1.6 Mb segment of the AZFc region. Deleted regions are shown as faded, and the possible combinations of inversion and recombination events are given.

Our study revealed a total of 29.4% of AZF regions deletions (Table 1) on the Y chromosome (Fig. 1A), in infertile men. Of these, non-allelic homologous recombination (NAHR) events accounted for 25.8%, which include the AZFb deletions of 3.5% (P5/proximal P1) (Fig. 1B), both AZFbc deletions of 2.3% (P4/distal P1 and P5/distal P1) (Fig. 1B), the complete AZFc deletions of 6.9% (b2/b4) (Fig. 1C,D), and the partial AZFc deletions of 13.1% [b1/b3 (2.7%) (Fig. 1E); gr/gr (5.1%) (Fig. 1FI,FII,FIII), b2/b3 (3.7%) (Fig. 1GI,GII,GIII); b3/b4 (1.5%) (Fig. 1HI,HII,HIII)]. The deletion of the AZFa region was observed in 31 infertile men (3.2%), consisting of 27 azoospermia (3.5%), 3 oligozoospermia (2.9%) and one oligoteratozoospermia man (1%) (Table 1). The deletion of AZFb region (P5/proximal P1) was detected in 34 infertile men (3.5%), of which 30 were azoospermic (3.9%) and 4 were oligozoospermic (3.8%) (Table 1). The deletions of both AZFbc regions (P4/distal P1 and P5/distal P1) with the absence of STSs markers in both AZFb and AZFc regions were identified in 22 azoospermic men (2.3%). A total of 67 infertile men (6.9%) showed deletion of the complete AZFc region (b2/b4) (Fig. 1C,D), of which 59 were azoospermic (7.7%), 6 were oligozoospermic (5.7%) and 2 were oligoteratozoospermic (2.1%) (Table 1). A very rare long Yq deletion removing all of the three AZFabc regions (absence of STSs markers in AZFa, AZFb and AZFc regions) was detected in 5 azoospermic men (0.5%). Importantly, none of these deletions AZFa, AZFb, AZFc, AZFbc or AZFabc was observed in the control men, signifying the importance of these AZF regions in spermatogenesis.

**Table 1:** Different types of deletion events observed at AZF locus in infertile and control men.

S:NO	Deletion Combination	Azoospermia (N = 771)					Oligozoospermia (N = 105)					Oligoteratozoospermia (N = 97)					Infertile (N = 973)					Fertile(N = 587)	
		No	%	^Chi2^	Odds	P	No	%	^Chi2^	Odds	P	No	%	^Chi2^	Odds	P	No	%	^Chi2^	Odds	P	No	%
**The AZFa, AZFb, AZFc, AZFbc and AZFabc deletions on Y chromosome**																							
1	AZFa	27	3.5	20.7	0.036	<0.0001	3	2.9	<5	0.029	0.12320	1	1	<5	0.010	0.5000	31	3.2	31.5	0.033	<0.0001	0	0
2	AZFb	30	3.9	23.36	0.040	<0.0001	4	3.8	<5	0.039	0.06071	0	0	0	0	0	34	3.5	34.6	0.036	<0.0001	0	0
3	AZFc (b2/b4)	59	7.7	46.96	0.083	<0.0001	6	5.7	<5	0.060	0.01451	2	2.1	<5	0.021	0.2487	67	6.9	69.39	0.074	<0.0001	0	0
4	AZFb,c	22	2.9	17.03	0.029	<0.0001	0	0	0	0	0	0	0	0	0	0	22	2.3	22.25	0.023	<0.0001	0	0
5	AZFab,c	5	0.7	>5	0.007	<0.0001	0	0	0	0	0	0	0	0	0	0	5	0.5	<5	0.005	<0.0001	0	0
	AZF deletions	143	18.7	157.6	0.228	<0.0001	13	12.4	14	0.141	8.22E-05	3	3.1	<5	0.032	0.1231	159	16.4	173.15	0.1953	<0.0001	0	0
**The AZFc Partial deletions**																							
**(a) The b1/b3 deletions**																							
6	b1/b30deletions of sY1191, sY1197, sY1161, sY1291, DAZ1 + 2,CDY1a	23	3	15.19	0.031	<0.0001	2	1.9	<5	0.02	0.06169	1	1	<5	0.01	0.2636	26	2.7	13.47	0.028	0.000242	1	0.17
**(b) The gr/gr deletions**																							
7	gr/gr (g1/g2; r1/r3), deletions of sY1291, DAZ1 + 2, CDY1a	25	3.2	7.37	0.034	0.00663	11	10.5	<5	0.12	3.4E-06	2	2.1	<5	0.02	0.3168	38	3.9	11.1	0.041	0.000863	6	1.02
8	gr/gr (r2/r4), deletions of sY1291, DAZ3 + 4, CDY1a	11	1.4	2.74	0.015	0.09786	1	0.9	<5	0.010	0.48304	0	0	0	0	0	12	1.2	2.01	0.013	0.156265	3	0.51
	gr/gr deletions	36	4.6	10.23	0.049	0.00138	12	11.4	<5	0.129	7.4E-06	2	2,1	<5	0.021	0.477252	50	5.1	13.1	0.054	0.000298	9	1.53
**(c) The b2/b3 inversion followed by gr/gr deletion or vise versa**																							
9	b2/b3 (g1/g3), sY1191, sY1206, DAZ3 + 4, CDY1a	10	1.3	<5	0.013	<0.0001	1	0.9	<5	0.010	0.15173	0	0	0	0	0	11	1.1	<5	0.0114	—	0	0
10	b2/b3 (r2/r3), DAZ1 + 2, CDY1a	17	2.2	8.4	0.023	0.00375	1	0.9	<5	0.010	0.39010	0	0	0	0	0	18	1.8	6.59	0.018	0.0103	2	0.34
11	b2/b3 (r1/r4), sY1191, DAZ3 + 4,CDY1a	7	1	<5	0.009	<0.0001	0	0	0	0	0	0	0	0	0	0	7	0.7	<5	0.007	—	0	0
	b2/b3_gr/gr_deletions	34	4.5	21.38	0.046	<0.0001	2	1.8	<5	0.019	0.11126	0	0	0	0	0	36	3.7	17.38	0.038	<0.0001	2	0.34
**(d) The b3/b4 inversion followed by gr/gr deletion or vise versa**																							
12	b3/b4, DAZ1 + 2, CDY1b	9	1.2	<5	0.012	<0.0001	0	0	0	0	0	0	0	0	0	0	9	0.9	<5	0.009	—	0	0
13	b3/b4, DAZ3 + 4, CDY1b	6	0.8	<5	0.008	<0.0001	0	0	0	0	0	0	0	0	0	0	6	0.6	<5	0.006	—	0	0
	b3/b4,_gr/gr_deletions	15	2	11.55	0.0198	0.00068	0	0	0	0	0	0	0	0	0	0	15	1.5	9.14	0.02	0.0025	0	0
The AZFc partial deletions (or) gr/gr deletions (gr/gr + b2/b3 and b3/b4 inversion followed by gr/gr deletions) (5.1 + 3.7 + 1.5 = 10.4)																	101	10.4	39.75	0.116	<0.0001	11	1.87
Total AZFc partial deletions (b1/b3 + gr/gr + b2/b3 and b3/b4 inversion followed by gr/gr deletions) (2.7 + 5.1 + 3.7 + 1.5 = 13.1)																	127	13.1	54.66	0.150	<0.0001	12	2.04
The total AZF deletions (both complete and partial deletions) (159 + 127) or (16.4 + 13.1) = 29.4%																	286	29.4	177.21	0.416	<0.0001	12	2.04

### Partial AZFc region deletions

We identified partial AZFc deletions in a total of 127 infertile (13.1%) and 12 fertile normospermic control men (2.0%), namely b1/b3 (Fig. 1E), gr/gr (Fig. 1FI,FII,FIII), b2/b3 (Fig. 1GI,GII,GIII), and b3/b4 (Fig. 1HI,HII,HIII) (Table 1). The b1/b3 deletion (partial AZFc deletion removing ~1.6 Mb DNA fragment) was detected in 26 (2.7%) infertile cases and one normospermic fertile man (0.2%) (Table 1). Of the 26 infertile men with b1/b3 deletions, 23 were azoospermic (3.0%), 2 were oligozoospermic (1.9%), one oligoteratozoospermic (1%) (Table 1), suggesting its importance in spermatogenesis.

The gr/gr (partial AZFc) deletion was detected in 50 infertile (5.1%) and 9 fertile control men (1.5%) (Fig. 1F; Table 1). The gr/gr deletion arises from 3 patterns of non-allelic homologous recombination events (NAHR) that occur between the amplicons g1/g2, r1/r3, and r2/r4. We detected 38 out of 50 infertile men (3.9%) and 6 out of 9 fertile men (1.02%) (Table 1) with the absence of the sY1291, *DAZ* cluster I (*DAZ*1 + 2), and *CDY1a*, which show the g1/g2 (Fig. 1FI) and r1/r3 (Fig. 1FII) NAHR patterns. Of the 38 infertile men, 25 were azoospermic (3.2%), 11 were oligozoospermic (10.5%) and 2 were oligoteratozoospermic (2%) (Table 1). The remaining 12 out of 50 infertile men (1.2%) and 3 out of 9 controls (0.5%) (Table 1) identified as gr/gr with the removal of sY1291, *DAZ* cluster II (*DAZ*3 + 4) and a copy of *CDY1a* show the r2/r4 NAHR pattern (Fig. 1FIII). Of the 12 infertile men, 11 were azoospermic (1.4%), and one oligozoospermic (0.9%) (Table 1).

Interestingly, we also observed two more inversions, b2/b3 (Fig. 1G) and b3/b4 (Fig. 1H) followed by gr/gr deletions or vice versa. The b2/b3 inversion (Fig. 1G) removes a 1.8 Mb segment of the AZFc region and was identified in 36 infertile cases (3.7%) and 2 controls (0.34%) (Table 1). The b2/b3 inversion was also found to follow 3 patterns of NAHR, which occur between the amplicons g1/g3, r2/r3, and r1/r4. The 11 (1.1%) out of 36 (3.7%) infertile men with b2/b3 deletions (consisting of 10 azoospermia 1.2% and one oligozoospermia 0.9%) (Table 1) show the g1/g3 NAHR pattern (Fig. 1GI). 18 out of 36 infertile (17 azoospermic and 1 oligospermic man) and 2 fertile control men (0.34%) with the b2/b3 deletion were identified with the r2/r3 (Fig. 1GII) NAHR pattern. The 7 azoospermic men out of 36 (0.7%) infertile men identified with the b2/b3 deletion show the r1/r4 NAHR pattern (Fig. 1GIII). However, interestingly the b2/b3 deletions with g1/g3 and r1/r4 NAHR patterns are completely absent among the sample of normospermic fertile control men (Table 1).

Another b3/b4 inversion (Fig. 1H) followed by a gr/gr deletion or vice versa, was found to remove a 1.6 Mb segment exclusively in 15 azoospermic men (1.5%). The b3/b4 inversion was also found to follow 3 patterns of NAHR, between the amplicons g1/g3, r1/r4 and r2/r3. The 9 infertile men (0.9%) identified showed two NAHR patterns: g1/g3 and r1/r4 (Fig. 1HI,HII). The remaining 6 out of 15 azoospermic men (0.6%) with the deletions of STS marker sY1291, *DAZ* cluster II (*DAZ*3 + 4) and a *CDY1b* gene, showed the r2/r3 (Fig. 1HIII) NAHR pattern. However, none of the control men showed the b3/b4 inversion (Table 1).

**Figure 2:**
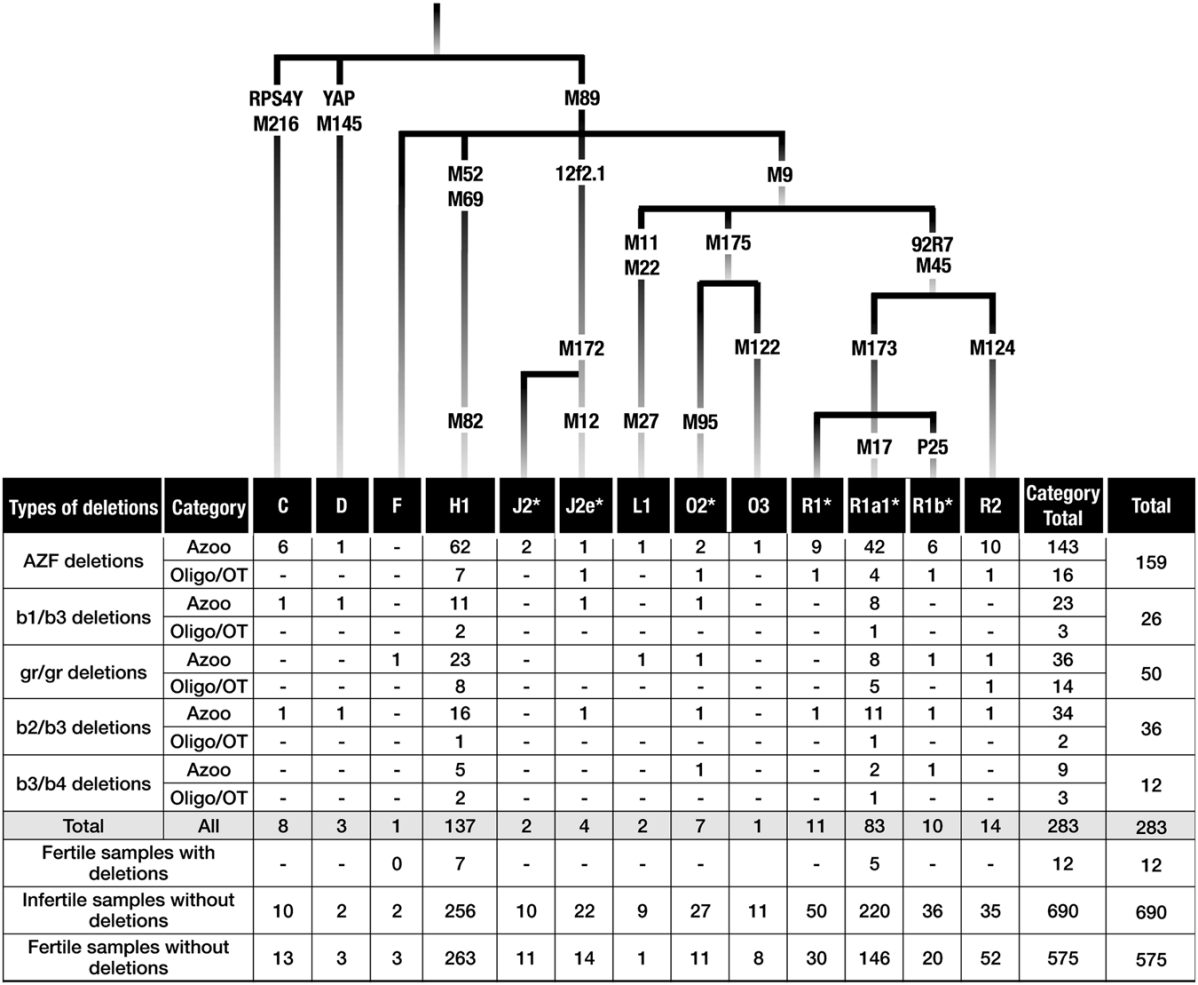
**Top:** Y-chromosomal phylogenetic tree showing the markers tested and the haplogroups they define. **Bottom:** Distributions and frequencies of haplogroups in fertile and infertile men with deletion and without deletions.

The haplogrouping of 973 infertile and 587 fertile control men, using Y chromosome binary markers^40,41^ revealed 8 distinct Y haplogroups in both cases and controls (Fig. 2). We compared the haplogroups of infertile men with or without AZF deletions and fertile control men; however, we failed to detect any specific deletion type that occurred only in a particular haplogroup background. We detected the two major haplogroups R1a-M17 and H1a-M82 with equal frequencies in both fertile and infertile men with/without deletions (Fig. 2). We also observed that the distributions of haplogroups were not different between the cases and the controls with/without deletions, suggesting that the haplogroups have no role in defining the deletion types and risk associations in infertile men.

## Discussion

Yq microdeletions are well-established causative factors for quantitative decline of spermatozoa and can lead to spermatogenic failure^46,47^. In the present study, we identified very high frequencies of classical Yq microdeletions of the AZF regions in infertile men, whereas no such deletion was observed among controls. This further strengthens the idea that the classical Yq microdeletions are a cause of spermatogenic failure in the Indian idiopathic infertile men. Our study revealed a very high frequency of deletion events (a total of 29.4%) in Indian infertile men, compared to other populations^4,12,48–56^. We observed 16.4% of classical Yq microdeletions, and these varied greatly in frequency among the populations, mainly due to the ethnic background, geographical region or case-control selection criteria^4,48–56^. The AZFc region is extremely rich in repetitive sequences and is organized as amplicons, and therefore a number of possible partial AZFc deletions (gr/gr, b1/b2, b2/b3, b3/b4) are proposed to be important risk factors for spermatogenic failure^15,19,20,26,27,29,37,42,57–62^. Interestingly, we identified AZFc partial deletions (gr/gr, b1/b3, b2/b3, b3/b4) in a total of 127 infertile men that accounted for 13.1%, with the impact of different *DAZ* and *CDY1* copies deletions and their associations leading to spermatogenic failure and male infertility. However, a few partial AZFc deletion studies have failed to show any such association with male infertility^28,31,63,64^.

In two meta-analyses, one consisting of seven studies reported significant association of gr/gr deletions with less motile sperm with low sperm count^65^, and another comprising of 18 case-control studies also established a strong relationship between gr/gr deletion and male infertility^66^. A few independent studies have also reported that the gr/gr deletion was more common among infertile men with azoo/oligozoospermia than in men with normozoospermia, suggesting that the deletion might be a significant risk factor for spermatogenic failure^30,32,58,67,68^. However, others failed to show any phenotypic impact of gr/gr deletions on spermatogenic failure^57,69,70^. Therefore, it is extremely important to study the frequencies of AZFc partial deletions and their association with fertility in Indian idiopathic infertile men. Our study showed that gr/gr deletions are more frequent among men with oligozoospermia **(**11.4%) than azoospermia (4.6%) and than in oligoteratozoospermia (2.1%); as expected, the prevalence is very low in controls (1.53%) (Table 1), suggesting that these partial deletions might also be a significant risk factor for spermatogenic failure in Indian idiopathic infertile men. Therefore, ethnic-specific differences in gr/gr deletion frequencies and their association with infertility are evident.

The previous studies have suggested that the gr/gr deletion frequency in the patient group was higher in the Asians (~10%) compared to the Europeans (~4.5%)^71^. Nevertheless, it is yet to be clarified whether the partial deletions (gr/gr, b1/b2, b2/b3, b3/b4) and their association is because of the lack of *DAZ* copies or due to other intervening genes that are also deleted. Some studies have suggested that the putative deletions of the *BPY*2 and *CDY1* genes were associated differentially with the distinct *DAZ* gene copy deletions that affect sperm pathology leading to infertility^25,72^. A few studies have also reported that the gr/gr deletion was neutral because of unknown compensatory mechanisms that had rescued the deleterious gr/gr deletion effect^25,42,60^.

Some individual reports have observed b1/b3 deletions in their population^15,73^. We in the present study detected 2.7% of b1/b3 deletions in Indian infertile men but 0.17% in controls, (P = 0.0002) (Table 1) suggesting a strong association of this deletion type with infertility in Indian idiopathic infertile men. Our study even detected b2/b3 (3.7%) and b3/b4 (1.5%) deletions including the deletion of two copies of *DAZ* and a copy of *CDY1*. The b2/b3 deletions removing 1.8 Mb DNA segment of AZFc region was reported in few studies^16,58,69,74–76^. The increased length of the b2/b3 deletion may raise the risk of complete AZFc deletion (b2/b4)^16,42^. We observed the b2/b3 deletion in 36 infertile men (3.7%) and 2 fertile men (0.34%), showing a (P < 0.0001) statistically significant difference between cases and controls (Table 1). The prevalence of the b2/b3 deletion in the present study was greater than reported previously in the Italian, Moroccan and North Indian populations^57,58,61,74^ but lower than in the Han-Chinese population (9.2%)^42,57,58,75^ or in Indian Dravidian men (7.21%)^73^.

Previous studies have reported that the gr/gr deletion was fixed in haplogroups D2b and Q1 in the Japanese and Chinese populations, respectively^15,37,42^. In the Northern Eurasian population, the b2/b3 partial deletion was fixed with haplogroup N^16^. However, some other studies have proposed that the b2/b3 deletion is different in different haplogroups^58,69,74^. Haplogrouping of 973 infertile and 587 normozoospermic fertile men with 24 Y chromosome binary markers^40,41^ in the present study revealed 8 major haplogroups, of which H1a-M82 and R1a-M17 were the two major haplogroups among both infertile and fertile men (Fig. 2). In India, the overall frequencies of haplogroups H1a-M82 and R1a-M17 were reported to be 40% and 17%, respectively^77^. Our results are also consistent with the general trend of Indian populations, where H1a-M82 is the most frequent haplogroup. These 8 haplogroups are common throughout India and are present among all the four major linguistic families^78^. Haplogroup H1a-M82 is an autochthonous haplogroup, whereas R1a-M17 is shared with the West Eurasian populations. Our previous studies have revealed that the people of Indian subcontinent are unique in their origin and differ significantly from the rest of the world in terms of their genetic affinities and disease susceptibility^79,80^. Therefore, heterogeneity in terms of the haplogroups observed among Indians and the Chinese are not surprising^80^.

Though our study included a substantial total sample size, subsamples such as oligozoospermic and oligoteratozoospermic patients remain small, which will have limited some statistical inferences. Even after the analysis of the Y chromosome partial deletions, the etiology remains unknown in a large proportion of the infertile men. Further analyses of genes, not only of the Y chromosome, but also of the X chromosome and the autosomes are required to understand the genetic causes of male infertility in a greater percentage of the idiopathic infertile cases.

## Conclusions

Our study revealed a very high frequency of AZF deletion events in Indian infertile men (29.4%) compared to other populations. We observed 16.4% of AZF region deletions exclusively in infertile men, consisting of AZFa (3.2%), AZFb (3.5%), AZFc (6.9%), AZFbc (2.3%) and AZFabc (0.5%). However, these deletion frequencies differ greatly in different populations, mainly due to the ethnic background/case-control selection criteria. We also identified partial AZFc deletions (gr/gr, b1/b3, b2/b3, b3/b4) in 127 infertile men (13.1%). Therefore, ethnic-specific differences in the AZFc partial deletion frequencies and their association with infertility are also evident. We found that gr/gr deletions are more frequent among oligozoospermic (11.4%), than azoospermic (4.6%) or oligoteratozoospermic (2.1%) patients, and that as expected the prevalence is very low in controls (1.53%), suggesting that these partial deletions might be a significant risk factor for spermatogenic failure (low sperm counts) in Indian idiopathic infertile men. Some studies have suggested that AZFc partial deletions are fixed in specific haplogroups. However, in the present study, we found that the distribution of haplogroups was not different between cases and controls with/without deletions, and that all deletions were rare in controls, suggesting that haplogroup has no role in determining risk associations in Indian infertile men. Thus, in our study we found some AZF deletion events that explained the infertility in these idiopathic infertile men. Indian populations are unique in their origin and have been practicing endogamy for the last two thousand years, and therefore it is important to add a study of the frequencies of AZF deletions on the Y chromosome and their association with fertility in Indian idiopathic infertile men to similar studies from other parts of the world.

## Materials and Methods

### Ethical statement and samples of infertile and fertile men

The Institutional Ethical Committees (IECs) of the participating institutes approved the study. The experiments were carried in accordance with the relevant guidelines and regulations approved for research on human samples. All the experimental protocols were approved by the IEC of the Centre for Cellular and Molecular Biology (CCMB). Before blood sample collection, the subjects underwent detailed medical and physical examinations. Informed written consents were obtained from all of 973 infertile and 587 fertile control men. The blood samples of 973 infertile men, consisting of 771 azoospermia (complete absence of sperm), 105 oligozoospermia (low sperm count) and 97 oligoteratozoospermia (low sperm count with abnormal shape and size) patients, were collected from the Genetic Clinic, Institute of Reproductive Medicine, Kolkata, India. The blood samples of the remaining 40 oligoteratozoospermic men were collected from the Infertility Institute and Research Centre, Hyderabad, India. In both the hospitals, a team of doctors (urologists and andrologists) performed detailed clinical investigations, which included semen analyses, and recorded complete case histories. In the hospitals, the blood samples were subjected to karyotyping and endocrinological assays, such as for follicle-stimulating hormone (FSH), luteinizing hormone (LH), testosterone (T), prolactin (PRL) and thyroid-stimulating hormone (TSH). Patients included in the study did not exhibit any obstructions, pelvic injury or major illness, karyotype abnormalities or endocrinological defects.

The 587 fertile normozoospermic control men with matched ethnic backgrounds had normal semen parameters (>20 × 10^6^ sperm/ml semen fluid with normal motility and morphology), according to the World Health Organization guidelines^81,82^, and normal levels of inhibin B, testosterone T, LH and FSH. They volunteered themselves to be included in this study as controls. About 5.0 ml of blood were collected from 537 and 50 infertile men from 2 hospitals: a) The Genetic Clinic, Institute of Reproductive Medicine, Kolkata, India and b) The Infertility Institute and Research Centre, Hyderabad, India, respectively. In addition, all the controls had fathered at least one child, each with proven paternity by STR-based DNA fingerprinting (Profiler Plus; Applied Biosystem, Foster City, USA), and were enrolled in the study after obtaining informed written consents. DNA was isolated from all the blood samples of infertile and fertile control men using the method published elsewhere^83^.

### Polymerase Chain Reaction (PCR)

Primer sequences of the STS, and SNV markers were obtained from (www.ncbi.nlm.nih.gov/entrez/) and synthesized using an ABI394 oligo-synthesizer (Perkin Elmer, Foster City, California, USA). The Polymerase Chain Reaction **(**PCR) was performed in 0.2 ml thin-walled tubes using 50 ng of DNA, 10 pM of the STS primers mentioned above, 100 μM dNTPs, 10X PCR buffer containing 1.5 mM MgCl_2_, and 2 units of AmpliTaq Gold (Perkin Elmer). Amplification was carried out in a MJ Research Thermal Cycler (Waltham, MA 02451, USA) using the amplification conditions: 94 °C for 5 minutes, 35 cycles at 94 °C for 45 seconds, 60 °C for 45 seconds and 72 °C for 1 minute, followed by the final extension at 72 °C for 5 minutes. The PCR products were size fractionated using 2% agarose gel electrophoresis and detected by staining with ethidium bromide.

### Mapping of the AZFa, AZFb, AZFc complete (b2/b4), and AZFc partial deletions (gr/gr, b1/b2, b2/b3, b3/b4 including DAZ and CDY1 gene CNVs)

**Figure 3:**
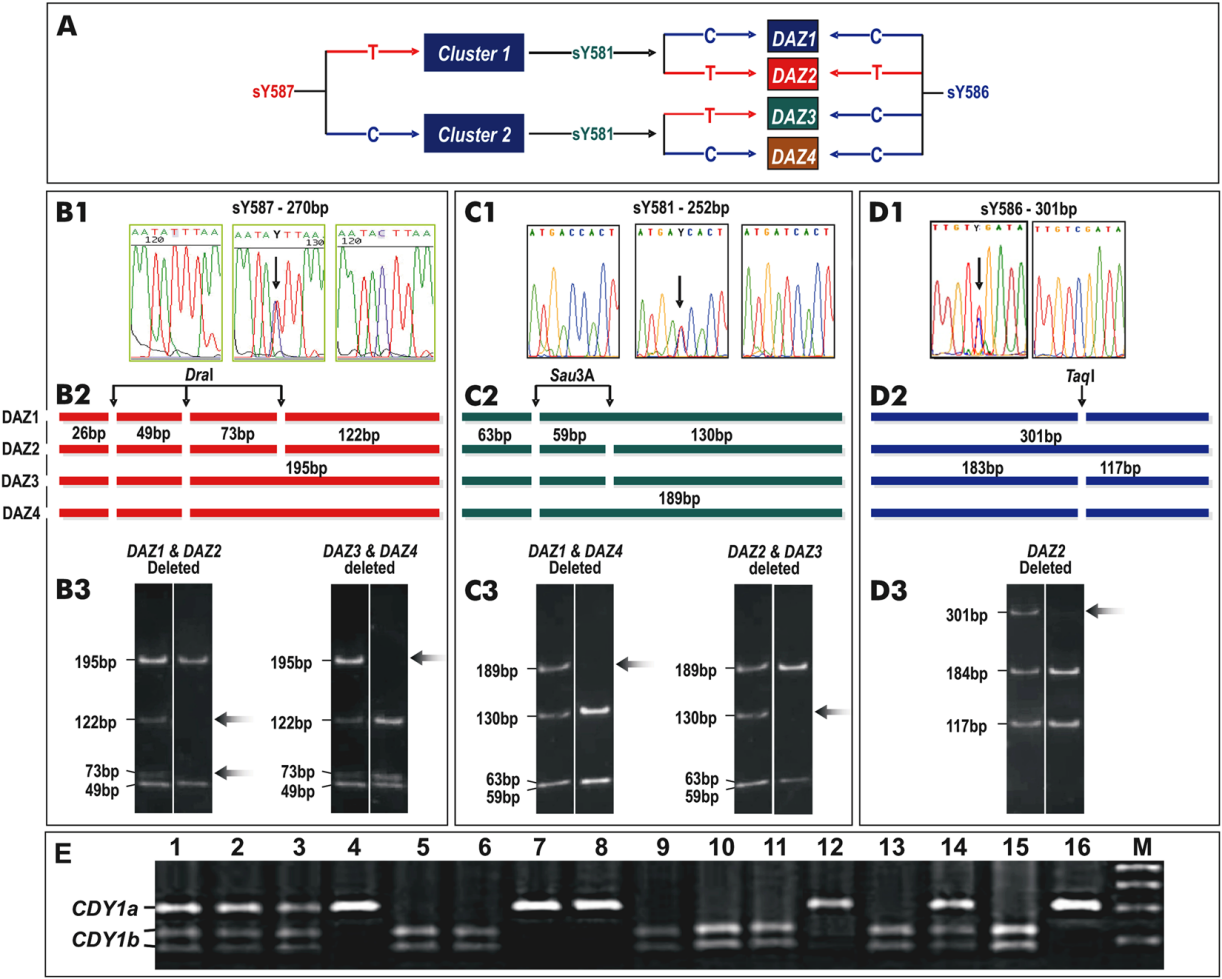
The *DAZ* and *CDY*1 genes copies deletions. (**A**) Schematic diagram showing the organization of *DAZ* gene copies. The 3 SNV markers sY587, sY581 and sY586 allow the differentiation of *DAZ* gene copies. (**B1)** The sequence electropherogram shows allele ‘T’ in the left panel, allele ‘C’ in the right panel and both the alleles in the middle panel. (**B2**) Schematic representation of the sY587 amplicon digested with restriction enzyme *Dra*I. (**B3**) The first lane of both panels is the digested amplicon of fertile controls. The second lane of the first panel shows the absence of the 73 bp and 122 bp fragments, suggesting the deletion of *DAZ*1/*DAZ*2. The second lane of the second panel shows the absence of the 195 bp fragment, suggesting the deletion of *DAZ*3/*DAZ*4. (**C1**) The sequence electropherogram shows allele ‘C’ in the left panel, allele ‘T’ in the right panel and both the alleles in the middle panel. (**C2**) Schematic representation of the sY581 amplicon digested with restriction enzyme *Sau*3A. (**C3**) The first lane of both panels is the digested amplicon of fertile men. The second lane of the first panel shows the absence of the 189 bp fragment, which suggests the deletion of *DAZ*1/*DAZ*4. The second lane of the second panel shows the absence of the 130 bp fragment, suggesting the deletion of *DAZ*2/*DAZ*3. (**D1**) The sequence electropherogram shows allele ‘C’ in the right panel and both ‘C’ and ‘T’ alleles in the left panel. (**D2**) Schematic representation of the amplicon of sY586 digested with *Taq*I. (**D3**) The first lane of the panel is the digested amplicon of fertile man. The second lane shows the absence of the 301 bp fragment, suggesting the deletion of *DAZ*2. (**E**) PCR-RFLP of *CDY*1-specific SNV; *CDY*1-7750 was amplified and digested with *Pvu*II and size-fractionated in a 2.0% agarose gel. *CDY1b* has a *Pvu*II restriction site, but *CDY*1a does not have a *Pvu*II restriction site. Lanes 1, 2, 3 and 14 – showing the intact *CDY*1a and the digested *CDY*1b. Lanes 4, 7, 8 and 12 – showing the presence of only *CDY*1a. Lanes 5, 6, 9, 10, 11, 13 and 15 – showing the presence of only *CDY*1b. Lane 16 undigested DNA and lane M marker.

The AZFa region deletion was detected using STSs markers: sY82, sY83, sY84, sY86, sY740, sY741, sY742, sY743, sY746, sY615, DBY and USP9Y. The AZFb deletion was identified by screening of STSs markers: sY98, sY100, sY113, sY121, sY124, sY127, sY128, sY130, sY134, sY142, sY143, sY145 and sY146. To define the AZFc complete (b2/b4), and partial (gr/gr, b1/b2, b2/b3) deletions, we used STSs markers: sY153, sY158, sY242, sY254, sY255, sY1258, sY1161, sY1197, sY1191, sY1291, sY1206 and sY1201 present within the amplicons. Further, to detect the presence/absence of particular *DAZ* gene copies in the AZFc region, we used multiple approaches. We first directly sequenced the PCR amplified products of following 3 additional STS markers sY587 (Fig. 3A,B1), sY581 (Fig. 3A,C1), and sY586 (Fig. 3A,D1), to detect the SNVs that differ between the copies of DAZ genes^23^. The DNA sequences of the sY587, sY581 and sY586 amplicons were aligned with the reference sequences (G63908, G63907, G63906) to detect the presence or absence of SNVs specific for *DAZ* gene copy/copies. Further, the DAZ copies were confirmed by digesting the sY587, sY581 and sY586 amplicons using *Dra*I (Fig. 3B2,B3), *Sau*3A (Fig. 3C2,C3) and *Taq*I (Fig. 3D2,D3), restriction enzyme digestions to detect the Restriction Fragment Length Polymorphism of PCR-Amplified Fragments (PCR-RFLP). Similarly, the *CDY1* gene copy/copies were amplified using the^28^ *CDY*1-specific SNV_*CDY*1-7750_[Primer pairs F: 5′ gaaatgccataatgtgctaacactg 3′; R: 5′ aaggagagtgttaatacataccctg 3′], the amplified products were then digested with *Pvu*II to detect the PCR-RFLP that differentiates *CDY*1a from *CDY*1b (Fig. 3E). The PCR-RFLP products of both the *DAZ* and *CDY1* genes copies were differentiated by size fractionation using 2% agarose gel electrophoresis and visualized.

### Southern hybridization

**Figure 4:**
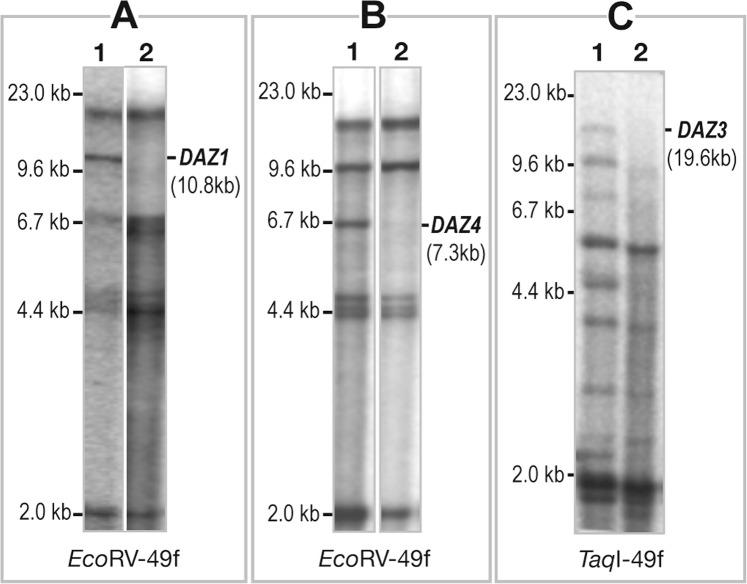
DAZ copy deletions confirmed by Southern hybridization analysis. (**A**) The first lane of the panel is the *Eco*RV-digested amplicon of fertile man. The second lane shows the *Eco*RV-digested DNA from an infertile man with a *DAZ1* deletion confirmed by hybridized with the 49f probe. (**B**) The first lane of the panel is the *Eco*RV-digested amplicon of fertile man. The second lane shows the infertile* Eco*RV-digested DNA from an infertile man with a *DAZ4* deletion confirmed by hybridized with the 49f probe. (**C**) The first lane of the panel is the *Eco*RV-digested amplicon of fertile man. The second lane shows the *Taq*I-digested DNA from an infertile man with a *DAZ3* deletion confirmed by hybridized with 49f probe. Although DNA of both fertile and infertile men were run in the same gel, transferred, and hybridized, they were not in the adjacent lanes. Therefore, we have cropped the images and placed the relevant adjacent for better comparison. We have provided the unedited autoradiogram as a Supplementary File.

Southern hybridization was carried out to further confirm the *DAZ* gene copy deletions, using representative DNA samples (2–4) of different deletion combinations of *DAZ* gene copies (wherever sufficient DNA was available) (Fig. 4). About 5.0 ug of DNA from chosen infertile men was digested separately with *Eco*RI and *Taq*I restriction enzymes. After completion of digestions, the DNA samples were size fractionated using 1.0% agarose gel and then transferred onto a Hybond N+ nylon membrane (Amersham Pharmacia, Buckinghamshire, United Kingdom) by capillary transfer, using 0.4 N NaOH. The membrane was further hybridized at 65 °C with a *DAZ*-specific hybridization probe 49f, radiolabeled with ^32^P (BRIT, Jonaki, India) in 0.5 M phosphate buffer and 7% SDS. After hybridization, excess probe was washed from the membrane with three changes of solution containing 2X SSC and 1% SDS at 65 °C for 45 minutes. Washed blots were exposed to a phosphor imager screen (Fuji, Japan) and images were acquired after 2 hrs (Fig. 4).

### Identification of Y-chromosomal haplogroups

All the infertile (973) and fertile control (587) men were haplogrouped using 24 Y chromosome binary markers^40,41^. PCR was carried out for all 24 binary markers, the amplified products were then directly sequenced using Sanger sequencing, and the haplogroups were assigned based on the sequence.

### Statistical analysis

The types of deletion combinations observed in our study were tabulated (Table 1), and comparisons were made between each category versus control using biostatistical tools available online (http://faculty.vassar.edu/lowry/VassarStats.html). To confirm the results, statistical tests were repeated at least twice. P values less than 0.05 were considered as statistically significant changes.

## Supplementary information

Supplementary Information

## References

[1] Lahn BT, Page DC (1997). Functional coherence of the human Y chromosome. Science.

[2] Krausz C, Degl’Innocenti S (2006). Y chromosome and male infertility: update, 2006. Frontiers in bioscience: a journal and virtual library.

[3] Vogt PH (1996). Human Y chromosome azoospermia factors (AZF) mapped to different subregions in Yq11. Human molecular genetics.

[4] Simoni M, Tuttelmann F, Gromoll J, Nieschlag E (2008). Clinical consequences of microdeletions of the Y chromosome: the extended Munster experience. Reproductive biomedicine online.

[5] Krausz C, Forti G, McElreavey K (2003). The Y chromosome and male fertility and infertility. International journal of andrology.

[6] Skaletsky H (2003). The male-specific region of the human Y chromosome is a mosaic of discrete sequence classes. Nature.

[7] Kamp C (2001). High deletion frequency of the complete AZFa sequence in men with Sertoli-cell-only syndrome. Molecular human reproduction.

[8] Sun C (2000). Deletion of azoospermia factor a (AZFa) region of human Y chromosome caused by recombination between HERV15 proviruses. Human molecular genetics.

[9] Sun C (1999). An azoospermic man with a de novo point mutation in the Y-chromosomal gene USP9Y. Nature genetics.

[10] Foresta C (2000). Role of the AZFa candidate genes in male infertility. Journal of endocrinological investigation.

[11] Foresta C, Ferlin A, Moro E (2000). Deletion and expression analysis of AZFa genes on the human Y chromosome revealed a major role for DBY in male infertility. Human molecular genetics.

[12] Krausz C (2014). EAA/EMQN best practice guidelines for molecular diagnosis of Y-chromosomal microdeletions: state-of-the-art 2013. Andrology.

[13] Kuroda-Kawaguchi T (2001). The AZFc region of the Y chromosome features massive palindromes and uniform recurrent deletions in infertile men. Nature genetics.

[14] Repping S (2002). Recombination between palindromes P5 and P1 on the human Y chromosome causes massive deletions and spermatogenic failure. American journal of human genetics.

[15] Repping S (2003). Polymorphism for a 1.6-Mb deletion of the human Y chromosome persists through balance between recurrent mutation and haploid selection. Nature genetics.

[16] Repping S (2004). A family of human Y chromosomes has dispersed throughout northern Eurasia despite a 1.8-Mb deletion in the azoospermia factor c region. Genomics.

[17] Repping S (2006). High mutation rates have driven extensive structural polymorphism among human Y chromosomes. Nature genetics.

[18] Blanco P (2000). Divergent outcomes of intrachromosomal recombination on the human Y chromosome: male infertility and recurrent polymorphism. Journal of medical genetics.

[19] Reijo R (1995). Diverse spermatogenic defects in humans caused by Y chromosome deletions encompassing a novel RNA-binding protein gene. Nature genetics.

[20] Habermann B (1998). DAZ (Deleted in AZoospermia) genes encode proteins located in human late spermatids and in sperm tails. Human reproduction.

[21] Lahn BT, Page DC (1999). Retroposition of autosomal mRNA yielded testis-specific gene family on human Y chromosome. Nature genetics.

[22] Lahn BT (2002). Previously uncharacterized histone acetyltransferases implicated in mammalian spermatogenesis. Proceedings of the National Academy of Sciences of the United States of America.

[23] Saxena R (2000). Four DAZ genes in two clusters found in the AZFc region of the human Y chromosome. Genomics.

[24] Ferlin A, Moro E, Rossi A, Foresta C (2001). CDY1 analysis in infertile patients with DAZ deletions. Journal of endocrinological investigation.

[25] Krausz C (2009). Phenotypic variation within European carriers of the Y-chromosomal gr/gr deletion is independent of Y-chromosomal background. Journal of medical genetics.

[26] Fernandes S (2002). High frequency of DAZ1/DAZ2 gene deletions in patients with severe oligozoospermia. Molecular human reproduction.

[27] Fernandes S (2004). A large AZFc deletion removes DAZ3/DAZ4 and nearby genes from men in Y haplogroup N. American journal of human genetics.

[28] Machev N (2004). Sequence family variant loss from the AZFc interval of the human Y chromosome, but not gene copy loss, is strongly associated with male infertility. Journal of medical genetics.

[29] Ferlin A (2005). Association of partial AZFc region deletions with spermatogenic impairment and male infertility. Journal of medical genetics.

[30] Giachini C (2005). The gr/gr deletion(s): a new genetic test in male infertility?. Journal of medical genetics.

[31] Hucklenbroich K (2005). Partial deletions in the AZFc region of the Y chromosome occur in men with impaired as well as normal spermatogenesis. Human reproduction.

[32] Lynch M (2005). The Y chromosome gr/gr subdeletion is associated with male infertility. Molecular human reproduction.

[33] de Vries JW (2002). Reduced copy number of DAZ genes in subfertile and infertile men. Fertility and sterility.

[34] de Vries JW (2002). Clinical relevance of partial AZFc deletions. Fertility and sterility.

[35] Vogt PH (2005). AZF deletions and Y chromosomal haplogroups: history and update based on sequence. Human reproduction update.

[36] Stouffs K (2008). Do we need to search for gr/gr deletions in infertile men in a clinical setting?. Human reproduction.

[37] Yang Y (2010). Differential effect of specific gr/gr deletion subtypes on spermatogenesis in the Chinese Han population. International journal of andrology.

[38] Lin YW (2005). Polymorphisms associated with the DAZ genes on the human Y chromosome. Genomics.

[39] Writzl K, Zorn B, Peterlin B (2005). Copy number of DAZ genes in infertile men. Fertility and sterility.

[40] Jobling MA, Tyler-Smith C (2003). The human Y chromosome: an evolutionary marker comes of age. Nature reviews. Genetics.

[41] Consortium YC (2002). A nomenclature system for the tree of human Y-chromosomal binary haplogroups. Genome research.

[42] Lu C (2009). The b2/b3 subdeletion shows higher risk of spermatogenic failure and higher frequency of complete AZFc deletion than the gr/gr subdeletion in a Chinese population. Human molecular genetics.

[43] Ferlin A (2007). Y chromosome haplogroups and susceptibility to testicular cancer. Molecular human reproduction.

[44] Jobling MA, Tyler-Smith C (2017). Human Y-chromosome variation in the genome-sequencing era. Nature reviews. Genetics.

[45] Carvalho CM (2003). Lack of association between Y chromosome haplogroups and male infertility in Japanese men. American journal of medical genetics. Part A.

[46] Kleiman SE (2007). Expression profile of AZF genes in testicular biopsies of azoospermic men. Human reproduction.

[47] Zhang YS (2014). Azoospermia factor microdeletions: occurrence in infertile men with azoospermia and severe oligozoospermia from China. Andrologia.

[48] Foresta C (1998). High frequency of well-defined Y-chromosome deletions in idiopathic Sertoli cell-only syndrome. Human reproduction.

[49] Ferlin A (2007). Male infertility: role of genetic background. Reproductive biomedicine online.

[50] Ferlin A (2007). Molecular and clinical characterization of Y chromosome microdeletions in infertile men: a 10-year experience in Italy. The Journal of clinical endocrinology and metabolism.

[51] Saliminejad K, Khorram Khorshid HR (2012). Contradictory results in “Yq microdeletions in infertile men from Northern India” by Mittal *et al*. (Ann. Genet. 47 (2004) 331–337). European journal of medical genetics.

[52] Saliminejad K (2012). Discrepancy in the frequency of Y chromosome microdeletions among Iranian infertile men with azoospermia and severe oligozoospermia. Genetic testing and molecular biomarkers.

[53] Sachdeva K, Saxena R, Majumdar A, Chadda S, Verma IC (2011). Use of ethnicity-specific sequence tag site markers for Y chromosome microdeletion studies. Genetic testing and molecular biomarkers.

[54] Sen S, Pasi AR, Dada R, Shamsi MB, Modi D (2013). Y chromosome microdeletions in infertile men: prevalence, phenotypes and screening markers for the Indian population. Journal of assisted reproduction and genetics.

[55] Suganthi R, Vijesh VV, Vandana N (2014). & Fathima Ali Benazir, J. Y choromosomal microdeletion screening in the workup of male infertility and its current status in India. International journal of fertility & sterility.

[56] Thangaraj K (2003). Y chromosome deletions in azoospermic men in India. Journal of andrology.

[57] Wu B (2007). A frequent Y chromosome b2/b3 subdeletion shows strong association with male infertility in Han-Chinese population. Human reproduction.

[58] Shahid M, Dhillon VS, Khalil HS, Sexana A, Husain SA (2011). Associations of Y-chromosome subdeletion gr/gr with the prevalence of Y-chromosome haplogroups in infertile patients. European journal of human genetics: EJHG.

[59] de Llanos M, Ballesca JL, Gazquez C, Margarit E, Oliva R (2005). High frequency of gr/gr chromosome Y deletions in consecutive oligospermic ICSI candidates. Human reproduction.

[60] Zhang F (2006). A frequent partial AZFc deletion does not render an increased risk of spermatogenic impairment in East Asians. Annals of human genetics.

[61] Giachini C (2008). Partial AZFc deletions and duplications: clinical correlates in the Italian population. Human genetics.

[62] Foresta C, Moro E, Ferlin A (2001). Y chromosome microdeletions and alterations of spermatogenesis. Endocrine reviews.

[63] Ravel C (2006). GR/GR deletions within the azoospermia factor c region on the Y chromosome might not be associated with spermatogenic failure. Fertility and sterility.

[64] de Carvalho CM (2006). Study of AZFc partial deletion gr/gr in fertile and infertile Japanese males. Journal of human genetics.

[65] Visser L (2009). Y chromosome gr/gr deletions are a risk factor for low semen quality. Human reproduction.

[66] Stouffs K, Lissens W, Tournaye H, Haentjens P (2011). What about gr/gr deletions and male infertility? Systematic review and meta-analysis. Human reproduction update.

[67] Bansal SK (2016). Gr/gr deletions on Y-chromosome correlate with male infertility: an original study, meta-analyses, and trial sequential analyses. Scientific reports.

[68] Yang L (2008). Abrogation of TGF beta signaling in mammary carcinomas recruits Gr-1+CD11b+myeloid cells that promote metastasis. Cancer cell.

[69] Zhang F (2007). Partial deletions are associated with an increased risk of complete deletion in AZFc: a new insight into the role of partial AZFc deletions in male infertility. Journal of medical genetics.

[70] Stahl PJ (2011). Diagnosis of the gr/gr Y chromosome microdeletion does not help in the treatment of infertile American men. The Journal of urology.

[71] Navarro-Costa P, Goncalves J, Plancha CE (2010). The AZFc region of the Y chromosome: at the crossroads between genetic diversity and male infertility. Human reproduction update.

[72] Vogt PH, Falcao CL, Hanstein R, Zimmer J (2008). The AZF proteins. International journal of andrology.

[73] Vijesh VV, Nambiar V, Mohammed SI, Sukumaran S, Suganthi R (2015). Screening for AZFc partial deletions in Dravidian men with nonobstructive azoospermia and oligozoospermia. Genetic testing and molecular biomarkers.

[74] Imken L (2007). AZF microdeletions and partial deletions of AZFc region on the Y chromosome in Moroccan men. Asian journal of andrology.

[75] Eloualid A (2012). Association of spermatogenic failure with the b2/b3 partial AZFc deletion. PloS one.

[76] Rozen SG (2012). AZFc deletions and spermatogenic failure: a population-based survey of 20,000 Y chromosomes. American journal of human genetics.

[77] Rai N (2012). The phylogeography of Y-chromosome haplogroup h1a1a-m82 reveals the likely Indian origin of the European Romani populations. PloS one.

[78] Chaubey G, Metspalu M, Kivisild T, Villems R (2007). Peopling of South Asia: investigating the caste-tribe continuum in India. BioEssays: news and reviews in molecular, cellular and developmental biology.

[79] Reich D, Thangaraj K, Patterson N, Price AL, Singh L (2009). Reconstructing Indian population history. Nature.

[80] Dhandapany PS (2009). A common MYBPC3 (cardiac myosin binding protein C) variant associated with cardiomyopathies in South Asia. Nature genetics.

[81] World Health Organization. Laboratory Manual for the Examination of Human Semen and Sperm-Cervical Mucus Interaction. 4th ed. (Cambridge: Cambridge University Press; 1999).

[82] World Health Organization. WHO Laboratory Manual For The Examination And Processing Of Human Semen, fifth edition, 1–287 (WHO press, 2010).

[83] Thangaraj K (2006). A to G transitions at 260, 386 and 437 in DAZL gene are not associated with spermatogenic failure in Indian population. International journal of andrology.

