# Supplementary material for: High frequencies of Non Allelic Homologous Recombination (NAHR) events at the AZF loci and male infertility risk in Indian men
Source: Sci Rep. 2019 Apr 18;9:6276. doi: 10.1038/s41598-019-42690-0 (PMC6472346; doi:10.1038/s41598-019-42690-0)
Supplement: Supplementary file 1 — Supplementary Information [file 41598_2019_42690_MOESM1_ESM.pdf]

High frequencies of Non Allelic Homologous Recombination (NAHR) events at the AZF loci and male infertility risk in Indian men

Deepa Selvi Rani[1], Singh Rajender[2], Kadupu Pavani[1], Gyaneshwer Chaubey[3], Avinash A. Rasalkar[1], Nalini J. Gupta[4], Mamta Deendayal[5], Baidyanath Chakravarty[4], Kumarasamy Thangaraj*[1]

[1]CSIR-Centre for Cellular and Molecular Biology, Hyderabad, India

[2]CSIR-Central Drug Research Institute, Lucknow, India

[3]Department of Zoology, Banaras Hindu University, Varanasi, India

[4]Institute of Reproductive Medicine, Salt Lake, Kolkata, India

[5]Infertility Institute and Research Centre, Hyderabad, India

Table S1. Clinical characteristics of the individuals with partial AZFc deletions

S.NO	Partial AZFc deletion Categories (numbers in parenthesis)	No of samples	Age (yrs)	LH (mIU/mL) 1.5-9.3	FSH (mIU/mL) 1.4-18.1	Testosterone (mIU/mL) 2.41-8.27	Testicular size (Left / Right)	Testicular pathology
1	b1/b3 (26)	23	25-40	NL	NL	NL	4cc/5cc -10cc/15cc	Azoospermia
		2	37-45	NL	NL	NL	4cc/5cc	Oligozoospermia
		1	39	NL	NL	NL	5cc/5cc	Oligoasthenospermia
2	gr/gr (50)	7	30-35	NL	NL	NL	1cc/2cc - 6cc/6cc	Azoospermia-Sertolicells only syndrome
		4	29-34	NL	NL	NL	1cc/3cc - 5cc/5cc	Azoospermia-MA
		5	19-33	NL	NL	NL	Absent/absent	Azoospermia-Soft rudimentary
		10	30-38	18-28	14-41	1-2.6	2cc/3cc - 5cc/6cc	Azoospermia
		4	33-37	NL	NL	NL	11cc/10cc - 20cc/15cc	Azoospermia-
		4	29-46	NL	NL	NL	3cc/2cc - 8cc-8cc	Azoospermia-
		2	29-34	NL	NL	NL	4cc/3cc - 5cc/4cc	Azoospermia-
		12	37-38	NL	NL	NL	4cc/5cc	Oligozoospermia
		2	39&40	NL	NL	NL	5cc/5cc	Oligoteratozoospermia
3	b2/b3(36)	15	28-35	13-32	23-60	0.9-2.4	1cc/1cc - 5cc/6cc	Azoospermia
		11	28-40	NL	NL	NL	1cc/1cc - 3cc/4cc	Azoospermia
		2	32&36	NL	NL	NL	3cc/4cc	Oligozoospermia
		8	29-46	NL	NL	NL	2cc/2cc - 8cc/10cc	Azoospermia
4	b3/b4(15)	7	29-46	NL	NL	NL	2cc/2cc - 8cc/10cc	Azoospermia
		8	28-45	NL	NL	NL	5cc/6cc - 10cc/10cc	Azoospermia

NL – Normal limit

Table S2. Sequence-tagged site (STS) markers and primer sequences used for detection of AZF loci deletion events on the Y chromosome

Regions	STS Markers	Forward primer sequences	Reverse primer sequences	Size of the amplicons
AZFa	sY82	ATCCTGCCCTTCTGAATCTC	CAGTGTCCACTGATGGATGA	264 bp
	sY83	CTTGAATCAAAGAAGGCCCT	CAATTTGGT TTGGCTGACAT	275 bp
	sY84	AGAAGGGTCTGAAAGCAG GT	GCCTACTACCTGGAGGCTTC	326bp
	sY86	GTGACACACAGACTATGCTTC	ACACACAGAGGGACAACCCT	320 bp
	sY746	GCTTCTCTGTAAAGTAGACACTGG	GGGAAATTGGGTTTTCCTAC	155bp
	sY740	ATGACTGGCTGTCGGAGTTC	AAGCTCTGTGGGAATGGTTG	100bp
	sY741	TGGCCTCCTTCTCTAGTCCA	TGGAGCTGACATGGGAAGA	97bp
	sY742	GGATTTCCCCACCTTGTTCT	TCCACATGTATCCCCTTGAA	95bp
	sY743	TTTGTGCATGAGAAAGCTGG	CTTGAAGTTAGCTGGGGTGC	100bp
	sY615	TAACTGCCTGCTTTTGCTCA	CAGACAAAGCCATCTGAAATAGG	130bp
	DBY	AGTTTATTCTAACCTAGG CAAACG	TCCAACCAGGCCTGTAGTGAGGCC	169bp
	USP9Y	CTTCACACAAATGCGTTTCA	TGCAATTATTTGAACAAACATGA	249 bp
AZFb	sY98	TGTCAGCAGGCTTAGTTCCT	CCTCTTCCCCACTACTTCAA	266bp
	sY100	TAAAGGAACTTCTGTGTGTAAACA	TAAGCCAGATAGGGGCTTCT	111bp
	sY113	GTTCTTTCCACAGCCCATAG	TGGAACACAATCCAAAATTG	304bp
	sY121	AGT TCA CAG AAT GGA GCC TG	CCT GTG ACT CCA GTT TGG TC	190bp
	sY124	CAG GCA GGA CAG CTT AAA AG	ACT GTG GCA AAG TTG CTT TC	109bp
	sY127	GGC TCA CAA ACG AAA AGA AA	CTG CAG GCA GTA ATA AGG GA	274bp
	sy128	GGATGAGACATTTTTGTGGG	GCCCAATGTAAAACTGGACA	228bp
	sy130	AGAGAGTTTTCTAACAGGGCG	TGGGAATCACTTTTGCAACT	173bp
	sY134	GTC TGC CTC ACC ATA AAA CG	ACC ACT GCC AAA ACT TTC AA	301bp
	sY142	AGCTTCTATTCGAGGGCTTC	CTCTCTGCAATCCCTGACAT	196bp
	sY143	GCAGGATGAGAAGCAGGTAG	CCGTGTGCTGGAGACTAATC	311bp
	sY145	CAA CAC AAA AAC ACT CAT ATACTCG	GGG CAT TGT ATG TTA ATA AGA GTT	125bp
AZFc	sy146	ACAAAAATGTGGCTCAGGGA	AAATAGTGTGCCCACCCAAA	173bp
	sY153	GCA TCC TCA TTT TAT GTC CA	ATG AGT CAC GAA AAC CCA AC	135bp
	sY158	CTCAGAAGTCCTCCTAATAGTTCC	ACAGTGGTTTGTAGCGGGTA	231bp
	sY242	ACA CAG TAG CAG CGG GAG TT3’	TCT GCC ACT AAA CTG TAA GCT CC	233bp
	sY254	GGG TGT TAC CAG AAG GCA AA	GAA CCG TAT CTA CCA AAG CAG C	400bp
	sY255	GTT ACA GGA TTC GGC GTG AT	CTC GTC ATG TGC AGC CAC	126bp
	sY1258	AACCCCATCTCTAGCAAAAATATG	TAGGTGACAGGGCAGGATTC	968bp
	sY1161	CGACACTTTTGGGAAGTTTCA	TTGTGTCCAGTGGTGGCTTA	330bp
	sY1197	TCATTTGTGTCCTTCTCTTGGA	CTAAGCCAGGAACTTGCCAC	453bp
	sY1191	CCAGACGTTCTACCCTTTCG	GAGCCGAGATCCAGTTACCA	385bp
	sY1291	TAAAAGGCAGAACTGCCAGG	GGGAGAAAAGTTCTGCAACG	527bp
	sY1206	ATTGATCTCCTTGGTTCCCC	GACATGTGTGGCCAATTTGA	394bp
	sY1201	CCGACTTCCACAATGGCT	GGGAGAAAAGTTCTGCAACG	677bp
	sY581	CACTGCCCTAATCCTAGCACA	TCTTCTGGACATCCACGTCA	252bp
	sY586	GTGTGGCACATATGCCTATAAA	TTGGTACATCCAGATGCAGAT	301bp
	sY587	TGGTTAATAAAGGGAAGGTGTTTT	TCTCCAGGACAGGAAAATCC	270bp

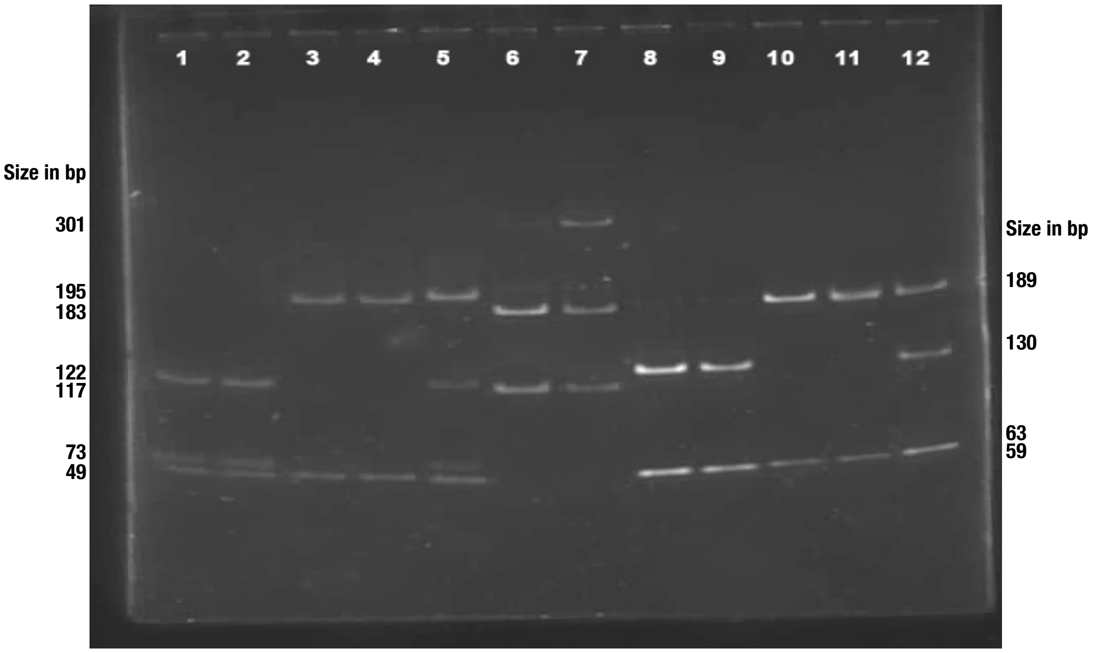

Figure S1. Deletions of DAZ copies confirmed by PCR-RFLP

The first two lanes 1 and 2 show the *DraI* (restriction enzyme) digested sY587 amplicons of infertile men. Absence of 195bp fragment suggests the deletion of *DAZ*3/*DAZ*4.

The third and fourth lanes show the *DraI* digested sY587 amplicons of infertile men. Absence of 122bp fragment suggests the deletion of *DAZ*1/*DAZ*2.

The fifth lane shows the *DraI* digested sY587 amplicon of fertile man containing all 4 fragments 49bp, 73bp, 122bp and 195bp, suggest the presence of all 4 DAZ copies.

The sixth lane shows the *Taq1* digested sY586 amplicon of infertile man containing 2 fragments of 117bp, 183bp and the absence of 301bp fragment suggest the deletion of DAZ2.

The seventh lane shows the *Taq1* digested sY586 amplicon of fertile man containing all 3 fragments of 117bp, 183bp and 301bp, suggest the presence of all 4 DAZ copies.

The eighth and ninth lanes show the *Sau3A* digested sY581 amplicon of two infertile men with the absence of 189 bp fragment, suggests the deletion of *DAZ*1/*DAZ*4

The tenth and eleventh lanes show the *Sau3A* digested sY581 amplicon of two infertile men with the absence of 130bp fragment, suggesting the deletion of *DAZ*2/*DAZ*3

The twelfth lane shows the *Sau3A* digested sY581 amplicon of fertile man containing all 4 fragments 189bp, 130bp, 63bp, 59bp, suggesting the presence of all 4 DAZ gene copies.

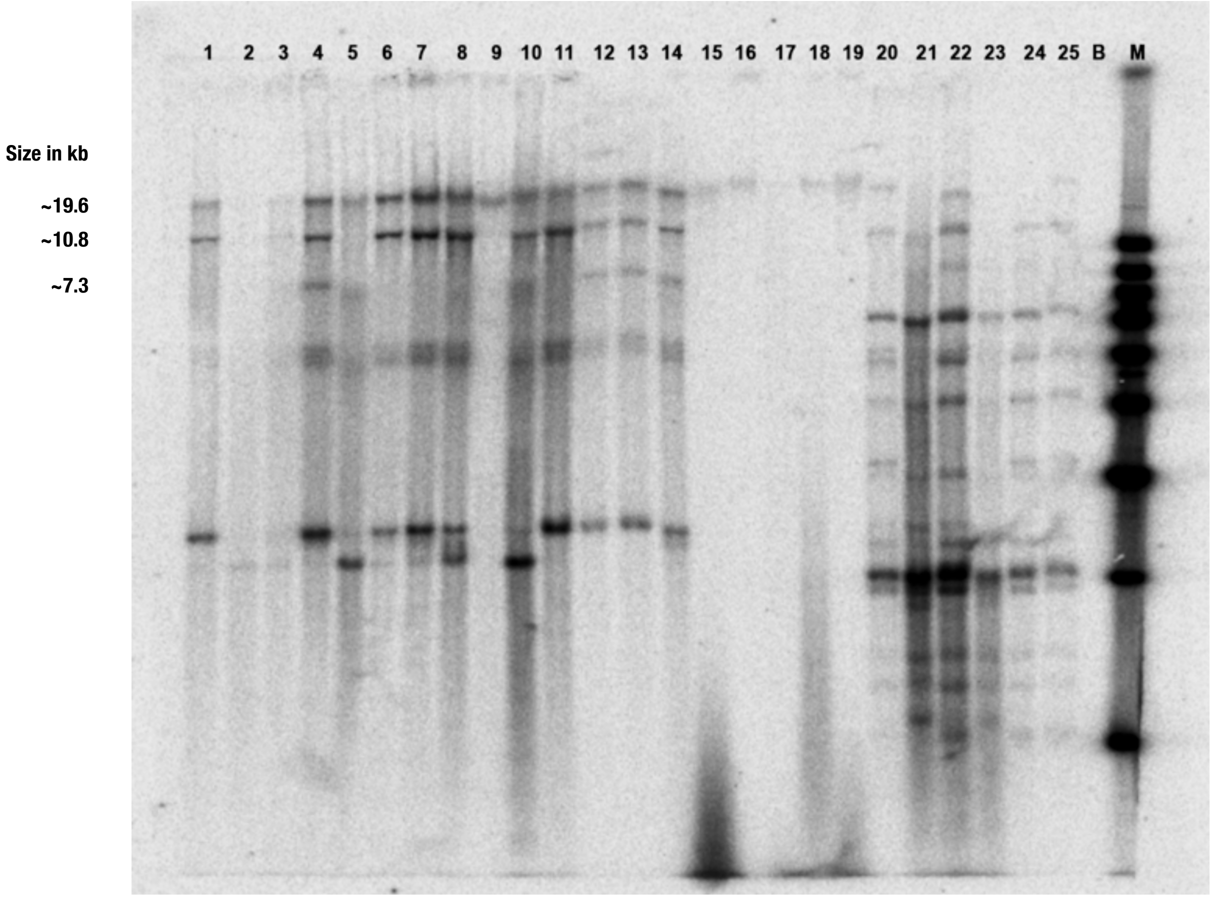

Figure S2. Original (unedited) autoradiograph of Southern hybridization confirms the deletion of DAZ copies. Lanes 1 – 19: genomic DNA digested with *EcoRV*; Lanes 20 – 25: genomic DNA digested with *Taq I*; B: blank, and M: DNA marker/ladder. Both *EcoRV* and *Taq I* digested genomic DNA of infertile men and fertile men were electrophoresed, transferred on to nylon membrane and hybridized with ^{32}P labeled probe 49f.

Lanes 1, 3, 6, 7, 8 and 11 are the genomic DNA of infertile men digested with *EcoRV*, showing the deletion of DAZ4 gene copy (~7.3kb). Lanes 5 and 9 are the genomic DNA of infertile men digested with *EcoRV*, showing the deletion of DAZ1 gene copy (~10.8kb). Lanes 4, 10, 12, 13 and 14 are the genomic DNA of fertile control men, showing the presence of both ~7.3kb and ~10.8kb DNA fragments.

Lanes 15, 16, 17, 18 and 19 were with insufficient amounts of DNA samples.

Lanes 21, 23 and 24 are the genomic DNA of infertile men digested with *Taq I*, shows the deletion of DAZ3 (~19.6kb).

Lanes 20 and 22 are the genomic DNA of infertile men and lane 25 is the genomic DNA of fertile men, showing the presence of 19.6kb DNA fragment.
